# Reply

**DOI:** 10.1002/hep4.2009

**Published:** 2022-05-20

**Authors:** Christian A. Hudert, Jake P. Mann

**Affiliations:** ^1^ Department of Pediatric Gastroenterology, Nephrology and Metabolic Diseases Charité Universitätsmedizin Berlin Berlin Germany; ^2^ Institute of Metabolic Science University of Cambridge Cambridge UK

To the editor,

We read with great interest the manuscript from Struwe et al.^[^ ^1^ ^]^ and their insightful comment on our recent article.^[^ ^2^ ^]^ Their X‐ray crystallography studies on mARC1 (encoded by MTARC1) illustrate two important general points: the limitation of *in silico* modeling and the challenge of understanding human variants.

*In silico* modeling of protein structure (and protein variants) is based on assumptions of amino acid interactions and how they combine in higher‐level protein folding. Despite enormous progress being made in the field,^[^ ^3^ ^]^ there is no replacement for the gold standard of X‐ray crystallography, as used by Struwe et al. For this reason, structural biology will continue to be indispensable for understanding mechanisms and drug discovery.

Together, these data suggest that it will be challenging to elucidate the mechanism through which this variant protects against liver disease. Its clinical effect has been well replicated but it appears to affect neither hepatic protein levels nor the structure of the protein.

It is often challenging to understand the mechanisms through which a specific missense variant confers disease. It has been 14 years since the effect of p.Ile148Met in PNPLA3 (patatin‐like phospholipase domain containing 3) was first described, and it has taken massive effort from the scientific community to understand its role in control of lipolysis.^[^ ^4^ ^]^

We look forward to the surprising and exciting results that will emerge over the next few years as the role of mARC1 in the liver is uncovered. Initial findings, such as *in silico* modeling, will be superseded by more robust observations like X‐ray crystallography, and supplemented by carefully conducted studies *in vitro* or *in vivo*. Through this method, we will collectively move closer to understanding how the p.Ala165Thr variant in mARC1 protects against fatty liver disease.

## AUTHOR CONTRIBUTIONS

*Manuscript draft and critical revision of the manuscript for important intellectual content*: Christian A. Hudert and Jake P. Mann.

## CONFLICT OF INTEREST

Nothing to report.

## FUNDING INFORMATION

Supported by the German Systems Biology Program “LiSyM” (31L0057 and 31L0058), sponsored by the German Federal Ministry of Education and Research; a Wellcome Trust fellowship (216329/Z/19/Z); a European Society for Pediatric Research Young Investigator Award; and a Children's Liver Disease Foundation Grant
